# Scrolling, Chatting, and Posting: Longitudinal Changes in Distinct Social Media Behaviors and Their Relationship With Psychological Distress and Mental Wellbeing in Adolescents

**DOI:** 10.1002/jad.70055

**Published:** 2025-09-24

**Authors:** S. Smout, T. Slade, E. Hunter, L. Thornton, L. A. Gardner, N. C. Newton, K. E. Champion, C. Chapman

**Affiliations:** ^1^ The Matilda Centre for Research in Mental Health and Substance Use University of Sydney Sydney Australia; ^2^ School of Medicine and Public Health, The University of Newcastle Newcastle New South Wales Australia; ^3^ School of Public Health, University of Sydney Sydney Australia

## Abstract

**Introduction:**

Over the past two decades, the prevalence of psychological distress and mental disorders among adolescents has markedly increased. This coincides with the advent and rapid adoption of social media, resulting in a proliferation of research examining time spent on social media and its relationship with mental health. However, to date, findings have been inconclusive. The active/passive model of social media behavior theorizes that “passive” social media behaviors (e.g., scrolling/watching) are associated with worse mental health outcomes than “active” behaviors (e.g., messaging or posting). The present study investigates both cross‐sectional and longitudinal relationships between active and passive social media behaviors and both psychological distress and mental wellbeing, while also examining differential effects of gender.

**Methods:**

This study uses data from two assessment waves (2021 and 2022) of a large adolescent Australian data set (*n* = 3205, T1 mean age 14.6 [SD: 0.62], 53.6% cisgender female/gender diverse). Three distinct behaviors were examined: (1) messaging/video calling friends (active), (2) posting content (active), and (3) scrolling or viewing content (passive).

**Results:**

There was little evidence of a longitudinal relationship between 12‐month change in any of the social media behaviors and psychological distress or mental wellbeing. While there were gender differences in the prevalence of the social media behaviors, there was no evidence of a gender interaction.

**Conclusions:**

Findings suggest the need to move beyond the active and passive model of social media behavior as a framework to explain the relationship between social media and adolescent mental health. We discuss several new directions for research and policy.

## 1 Introduction

Over the past two decades, the prevalence of psychological distress and mental disorders among adolescents has markedly increased (Piao et al. 2022). Large studies in the United States (Keyes et al. 2019; Daly 2022), Canada (Wiens et al. 2020), the United Kingdom (Cybulski et al. 2021) and Australia have all replicated these trends, with the steep rise commencing around 2007−2008. This coincides with the advent and rapid adoption of smartphones and social media; 89% of adolescents own a smartphone (Abi‐Jaoude et al. 2020) and in Australia, 97% currently access social media (Smout et al. 2021). The parallel timing of these trends has led to a proliferation of research examining the associations between screen time and adolescent mental health. Early work in this area, which relied on self‐reported measures of screen time and social media use, demonstrated an association between higher amounts of time spent on smartphones and higher levels of depression and psychological distress (Abi‐Jaoude et al. 2020; Twenge and Campbell 2018; Woo et al. 2021). However, several rigorous systematic reviews and meta‐analyzes have resulted in inconclusive findings (Ferguson et al. 2022; Tang et al. 2021; Odgers et al. 2020; Dickson and Kwan 2018).

Moving beyond time spent on screens in general, research has sought to understand the adolescent mental health outcomes of time spent on social media, with some studies reporting associations with anxiety, depression and self‐esteem (Karim et al. 2020; Gupta et al. 2022; Blomfield Neira and Barber 2014). The term “social media” refers to online social networks that allow users to create and share content, generally via comments, messages, photos and videos (Commissioner 2025). Recognizing that time spent on social media is a blunt measure on its own—as adolescent social media behaviors often vary widely—recent research has sought to differentiate the mental health outcomes of “active” versus “passive” social media behavior (Verduyn et al. 2017; Chen et al. 2019; Valkenburg et al. 2021). Active social media behavior includes posting, messaging, and interacting with other users, whereas passive behavior includes viewing content or scrolling social media feeds without interacting (Thorisdottir et al. 2019). The “active/passive model” proposes that passive behavior negatively impacts wellbeing through upward social comparison and envy, while active behavior leads to beneficial effects on wellbeing because it evokes social support and positive feedback (Valkenburg et al. 2021). While intuitively appealing, to‐date, research examining the mental health outcomes of active/passive social media behavior has yielded mixed results (Verduyn et al. 2017; Kim et al. 2020; Jensen et al. 2019; Beyens et al. 2020; Boer et al. 2022; Tibbs et al. 2025). Proponents of this model have highlighted the need for longitudinal research, particularly among general community samples of adolescents, to fully understand impacts of different social media behaviors on mental health and wellbeing (Verduyn et al. 2021).

Gender plays an important role in understanding psychological distress and wellbeing, as well as social media behavior in adolescents. Among adolescents aged 11−17 in Australia, females are more likely to report “high” or “very high” psychological distress (16% and 9.5%) compared to males (10% and 4%) (Health and Welfare 2021), and also have higher rates of anxiety, depression and low self‐esteem (Brennan et al. 2021). Gender is also associated with differing social media behavior (Yau and Reich 2019), with females using social media more, and in different ways than males (Twenge and Martin 2020). Additionally, females place greater emphasis on social relationships and are more inclined to seek feedback and engage in upward social comparison than males, suggesting that females may experience greater negative impacts of social media behavior (Twenge and Martin 2020). To better understand this gender difference, research has considered the “active/passive” dichotomy among males and females, revealing some interesting findings (Thorisdottir et al. 2019; Svensson et al. 2022). For example, an Icelandic study of adolescents aged 14−16 found that females both actively (e.g., posting photos and/or videos) and passively use social media more than males per day (Thorisdottir et al. 2019). This study also found that passive social media behavior was associated with emotional distress across both genders, however the relationship was more significant among females (Thorisdottir et al. 2019). Another study found that self‐presentation (i.e., posting information about oneself on social media) was strongly associated with internalising symptoms in females, however also revealed a negative association between online sociability (i.e., non‐private online communication) and internalising symptoms in males (Svensson et al. 2022). However, these studies are limited by cross‐sectional design, precluding any conclusions around whether a change in active or passive social media behaviors may be differentially associated with improved or worsened mental health by gender.

The present study aims to extend existing research in three important ways. First, we examine distinct social media behaviors and their associations with mental health and investigate cross‐sectional and longitudinal associations, allowing for both single‐timepoint frequency of social media use and within‐person *change* in social media behaviors as predictors of mental health outcomes. Thirdly, we investigate the role of gender in these relationships. Specifically, the present study utilizes data from a large cohort of Australian adolescents measured 12 months apart in 2021 and 2022 to examine: (1) The prevalence and 12‐month changes in three distinct social media behaviors—messaging/video calling friends, posting content, and passively viewing content—including differences by gender; (2) Cross‐sectional, longitudinal prospective, and longitudinal change‐based associations between social media behaviors and psychological distress and mental wellbeing; and (3) Interaction effects and subgroup analyzes by gender.

## 2 Methods

### 2.1 Participants

This paper is a secondary analysis of data collected from 3205 participants at 24‐month follow‐up (2021, Year 9) and 36‐month follow‐up (2022, Year 10) of the *Health4Life* study, a cluster randomized controlled trial conducted in 71 secondary schools across three Australian states (New South Wales, Queensland, and Western Australia) between 2019 and 2022. The Health4Life trial included an intervention delivered in Grade 7 (2019) targeting lifestyle behavior change (including screen time). However, previous analyzes have shown that the intervention was not effective in achieving lifestyle behavior change at any timepoint and was only effective immediately post‐intervention (2019) in addressing psychological distress and depression symptoms (Champion et al. 2023; Smout, Champion, O'Dean, Teesson, et al. 2024). As such, both control and intervention groups were included in the present study to maximize sample size, but intervention group allocation was adjusted for in all models (see Statistical Analysis for further information).

Ethics approval was granted by the Human Research Ethics Committees of the University of Sydney (2018/882), Curtin University (HRE2019‐0083), the University of Queensland (2019000037), the NSW Department of Education (SERAP no. 2019006), and the associated ethics committees for each participating school. Students provided active written consent and parents provided either active (written or verbal) or passive (opt‐out) consent. All consenting students completed online, self‐report surveys either in class time, or in their own time if they were away on the day of allocated class time. Additional details including sample size calculations, recruitment and further detail on consent procedures can be found in the published primary outcomes paper and study protocol (Champion et al. 2023; Teesson et al. 2020).

### 2.2 Measures

#### 2.2.1 Social Media Behaviors

At the time of survey development in early 2021, we consulted the literature to identify validated social media behavior measures and determined that—due to the rapid rate of technology change and differing hypotheses around social media—most studies developed their own bespoke measures of social media behavior and none of these were suitable to discern more social/interactive active behavior (messaging/video calling friends) and less social/interactive active behavior (posting). Further, many scales specified set platforms in the question wording, which would limit the generalizability of findings as new platforms emerge (Beyens et al. 2020; Coyne et al. 2021; Boers et al. 2020, 2019; Kaur et al. 2020; Sanders et al. 2019). Further information on the measures considered is available in Supporting Information S1: Note 1. As such, we developed a bespoke measure of social media behaviors that balanced feasibility (we required a short measure due to the time constraints of survey completion during school class time) and relevance (focussing on different behaviors rather than devices or platforms). The full measure is available in Supporting Information S1: Table 1 and is most similar to the measure developed by Beyens et al (2020, Supporting Information S1: Note 1). Briefly, students reported how frequently they (1) message or video call friends (on platforms such as Snapchat, Instagram, FaceTime, Whatsapp, text message etc), (2) post content on social media (stories, feed posts, videos etc), (3) view other peoples' content on social media (watching videos, scrolling through posts and stories etc). Students reported behaviors separately by weekend and weekday, recognizing that some schools and/or parents impose phone bans on school days. Frequency categories for weekdays were: “every day,” “3 to 4 days a week,” “1 to 2 days a week” and “not usually/not at all” and for weekends were: “both days,” “one day” and “not usually/not at all.” Weekend and weekday measurements were analyzed separately, except when examining change between the two timepoints. In this case weekday and weekend measurements were combined to create an overall change in weekly social media behavior. Change in frequency of each of the three behaviors between the two timepoints (henceforth referred to as T1 and T2) was calculated and categorized as: Large decrease (a decrease in frequency of 3 or more days a week); small decrease (a decrease of less than 3 but more than 0 days a week); no change; small increase (an increase of more than 0 but less than 3 days a week); and large increase (an increase of 3 or more days a week). The no change category was chosen as the reference category. Supporting Information S1: Note 2 contains more information about how these two variables were combined and how change was categorized.

#### 2.2.2 Psychological Distress

Participants completed the Kessler 6‐item scale (K6), reporting past 30‐day symptoms of psychological distress (Kessler et al. 2002). This summed K6 score ranges from 0 to 24, which was then dichotomized into the presence or absence of high psychological distress, indicated by scores of 13 or higher (Mewton et al. 2016; Kessler et al. 2010). High psychological distress is not disorder‐specific but indicates a probable mental illness. The K6 (and these associated cut‐offs) is widely used and has demonstrated excellent internal consistency in the current data (Cronbach's alpha = 0.91) and good predictive validity in adolescents (Mewton et al. 2016; Kessler et al. 2010).

#### 2.2.3 Mental Wellbeing

The Short Warwick–Edinburgh Mental Well‐being Scale (SWEMWBS) was used to measure mental wellbeing. The seven‐item scale asks participants to reflect on the past 2 weeks and report how frequently they were feeling: optimistic about the future, useful, relaxed, close to others, as well as dealing with problems well, feeling close to other people, and being able to make up their own mind about things. Frequency ranged from “None of the time” to “All of the time,” each converting to a score of 1−5 then summed to a total score out of 35, which is then converted to a metric score. A systematic review on the measurement of wellbeing among adolescents recommended the SWEMWBS (Rose et al. 2017), and it has been validated among Australian adolescents (Hunter et al. 2015). In the current data this scale has excellent internal consistency (Cronbach's alpha = 0.92).

#### 2.2.4 Sedentary Recreational Screen Time

The International Sedentary Assessment Tool was used to measure the amount of time students typically spent engaging in sedentary activity on weekdays and weekend days in the past week (Prince et al. 2017). For these analyzes, sedentary recreational screen time was included as a covariate given its association with social media use behaviors and to capture discrete findings related to the “active/passive” SMU dichotomy. Two items were used to calculate sedentary recreational screen‐time. Students reported time typically spent sitting, reclining or lying down while (1) “watching television or videos during your free time (including watching TV, DVDs, Netflix or online videos)” and (2) “using an electronic device during your free time (e.g., computers, laptops, Xbox, PlayStation, iPads or other tablets, smartphones, YouTube, Facebook or other social media, and the Internet).” In instances of dual screen use, students were instructed to only report time against one behavior to avoid double‐counting. Students' average daily sedentary recreational screen time was calculated as a weighted average of weekend and weekday time spent across these two categories of activity.

#### 2.2.5 Gender

The best‐practice two‐step approach to categorizing gender was used, whereby gender identity and sex assigned at birth are combined to identify cisgender male and female participants (those whose gender identity is congruous with their sex at birth) and transgender and gender diverse participants (those whose gender identity is different to their sex assigned at birth) (Kidd et al. 2022). Due to small numbers of gender diverse participants (*n* = 89, 2.8% of total sample), cisgender females and gender diverse participants were combined into one group, informed by prior literature that suggests cisgender female and gender diverse adolescents both have higher social media behavior than cisgender male adolescents (Smout, Champion, O'Dean, Halladay, et al. 2024). Gender was assessed at T1 (2021).

#### 2.2.6 Relative Family Affluence

Relative family affluence was included as a covariate to account for socioeconomic differences that may influence both technology access and mental health (Bohnert and Gracia 2023) among adolescents and was identified using the Family Affluence Scale III (FASIII). The FASIII has demonstrated good test−retest reliability (*r* = 0.9) and strong correlation with parental report (Torsheim et al. 2016). As parental income and education are often not known by children and adolescents, the FASIII utilizes proxy indicators of familial wealth (e.g., number of computers, number of bathrooms in home, etc) and a summed score is generated (Torsheim et al. 2016). The total score is then transformed into a normally distributed ridit score, which represents a participant's relative family affluence position within the overall sample (bottom 20%, middle 60%, top 20%).

### 2.3 Statistical Analysis

The frequency of each social media behavior at each timepoint and the change in each behavior across the two timepoints was calculated in the total sample, and separately for males and females/gender diverse participants. The association between each predictor (the three social media behaviors) and each mental health outcome (high psychological distress and high wellbeing) was separately examined in three logistic regression models: (1) cross‐sectional models examined the relationship between T2 measurements of both the predictor and outcome, (2) longitudinal prospective models examined the relationship between T1 measurement of the predictor and T2 measurement of the outcome, and (3) longitudinal change‐based models examined the relationship between change in the predictor over the two timepoints and T2 measurement of the outcome. All models controlled for gender (cisgender males, cisgender females and gender diverse participants), T2 relative family affluence (lower 20%, middle 60%, upper 20%), residing Australian state at the beginning of the trial (New South Wales, Queensland, Western Australia), intervention group allocation at the beginning of the trial (intervention, control) and T2 sedentary recreational screen time (total number of hours per day). Additionally, longitudinal prospective and change‐based models also controlled for the T1 outcome. Cluster‐robust standard errors were calculated to account for the clustering of individuals within schools. Gender differences in associations between predictors and outcomes were examined by adding a term representing the interaction between gender and each predictor. The overall main effect of the primary predictor variable in each model was assessed with a chi‐squared statistic. To adjust for multiple testing (i.e., two outcome variables) the *p* value representing a significant main effect was adjusted to 0.05/2 = 0.025. All analyzes were carried out using Stata 18.0 (StataCorp 2023).

## 3 Results

### 3.1 Sample Characteristics

The sample consisted of 3205 adolescents (53.6% cisgender female/gender diverse) with a mean age of 14.6 (SD: 0.62) at T1 and 15.7 (SD: 0.66) at T2. Most students (53%) attended independent schools, while 28% attended public schools, and 19% attended systemic catholic schools. Overall, 18% of participants fell in the lowest quintile of the population according to family affluence, 12% in the highest and the remainder (70%) in the middle three quintiles.

### 3.2 Prevalence of Social Media Behaviors

**Figure 1:**
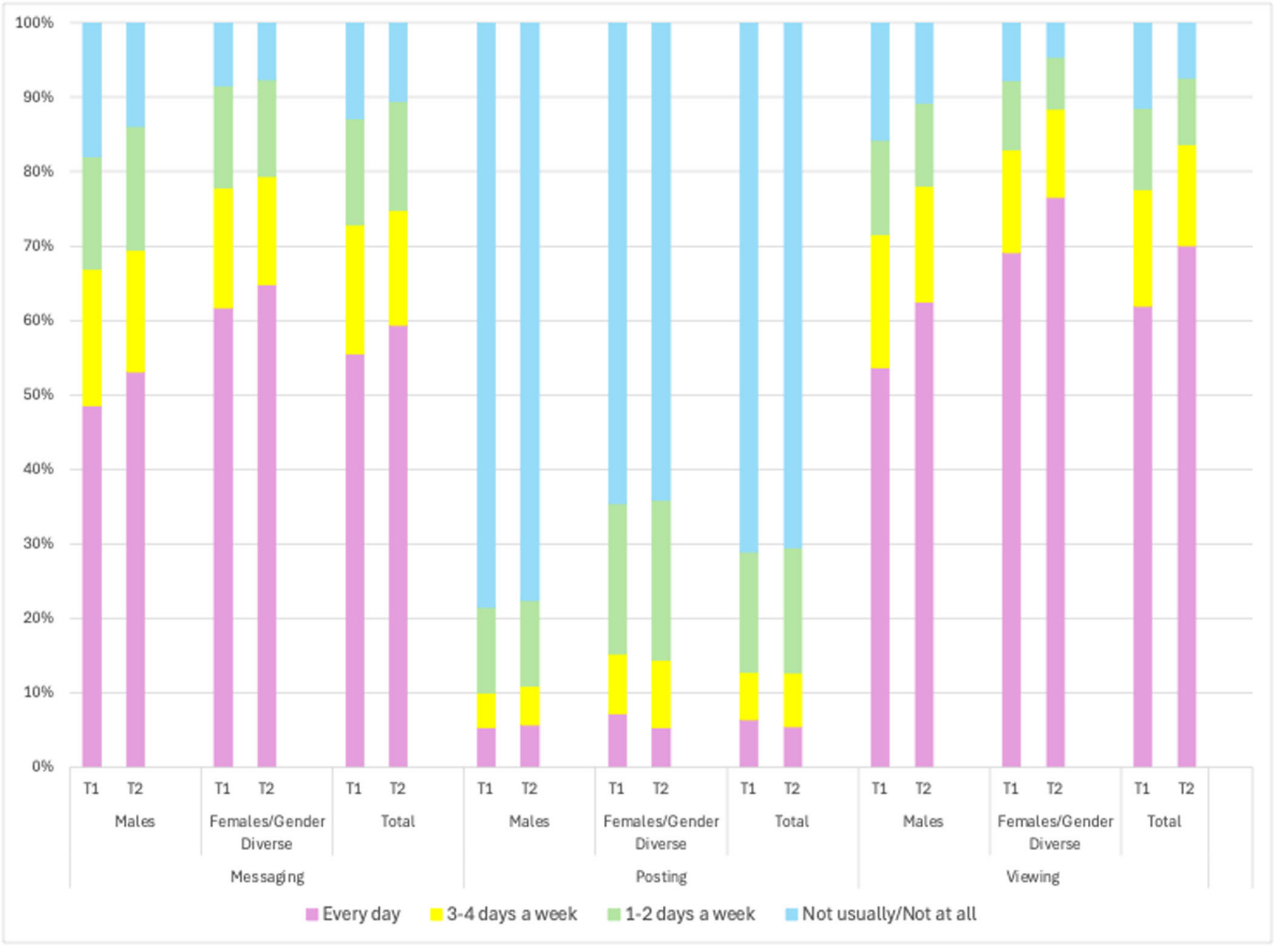
Weekday frequency of each social media behavior by timepoint and gender.

**Figure 2:**
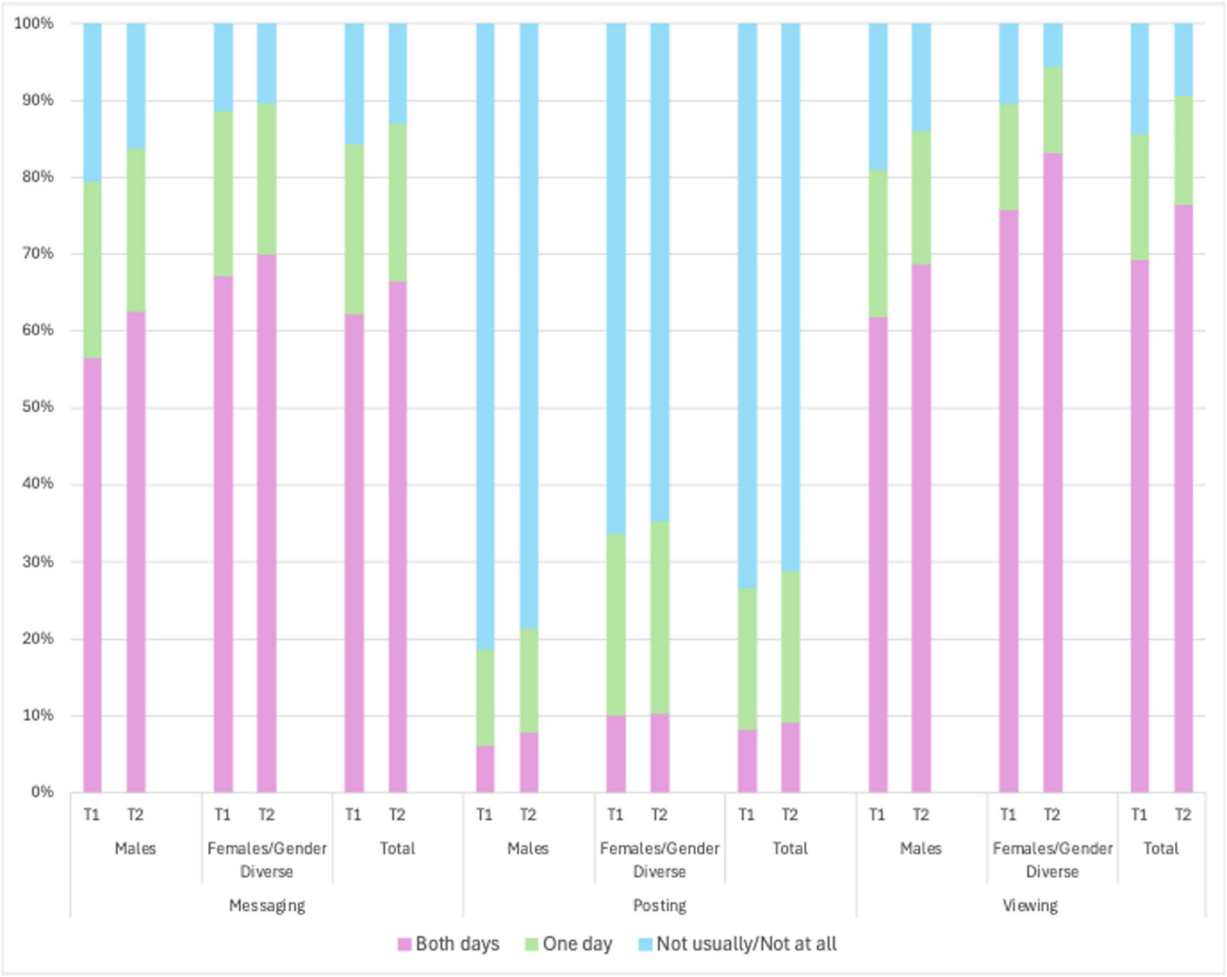
Weekend frequency of each social media behavior by timepoint and gender.

The prevalence and frequency of the three distinct social media behaviors is summarized by timepoint and gender in Figure 1 (weekdays) and Figure 2 (weekends). More than half of participants reported messaging or video calling friends every day on both weekdays and weekends (weekdays: 55.6% in T1 and 59.4% in T2; weekends: 62.3% in T1 and 66.6% in T2). Posting content was much less frequent, with less than 10% of participants reporting daily posting on both weekdays and weekends (weekdays: 6.4% in T1 and 5.5% in T2; weekends: 8.3% in T1 and 9.2% in T2). Viewing other peoples’ content was the most frequent behavior, again with most participants reporting that they did so daily on both weekdays and weekends (weekdays: 61.9% at T1 and 70.1% at T2; weekends: 69.4% in T1 and 76.5% in T2).

All three behaviors were more frequent (*p* < 0.001) among cisgender female and gender diverse participants compared to cisgender male participants on both weekdays and weekends at both timepoints (Figure 1 and 2, Supporting Information S1: Table 2).

### 3.3 Changes Over Time in Social Media Behaviors

**Figure 3:**
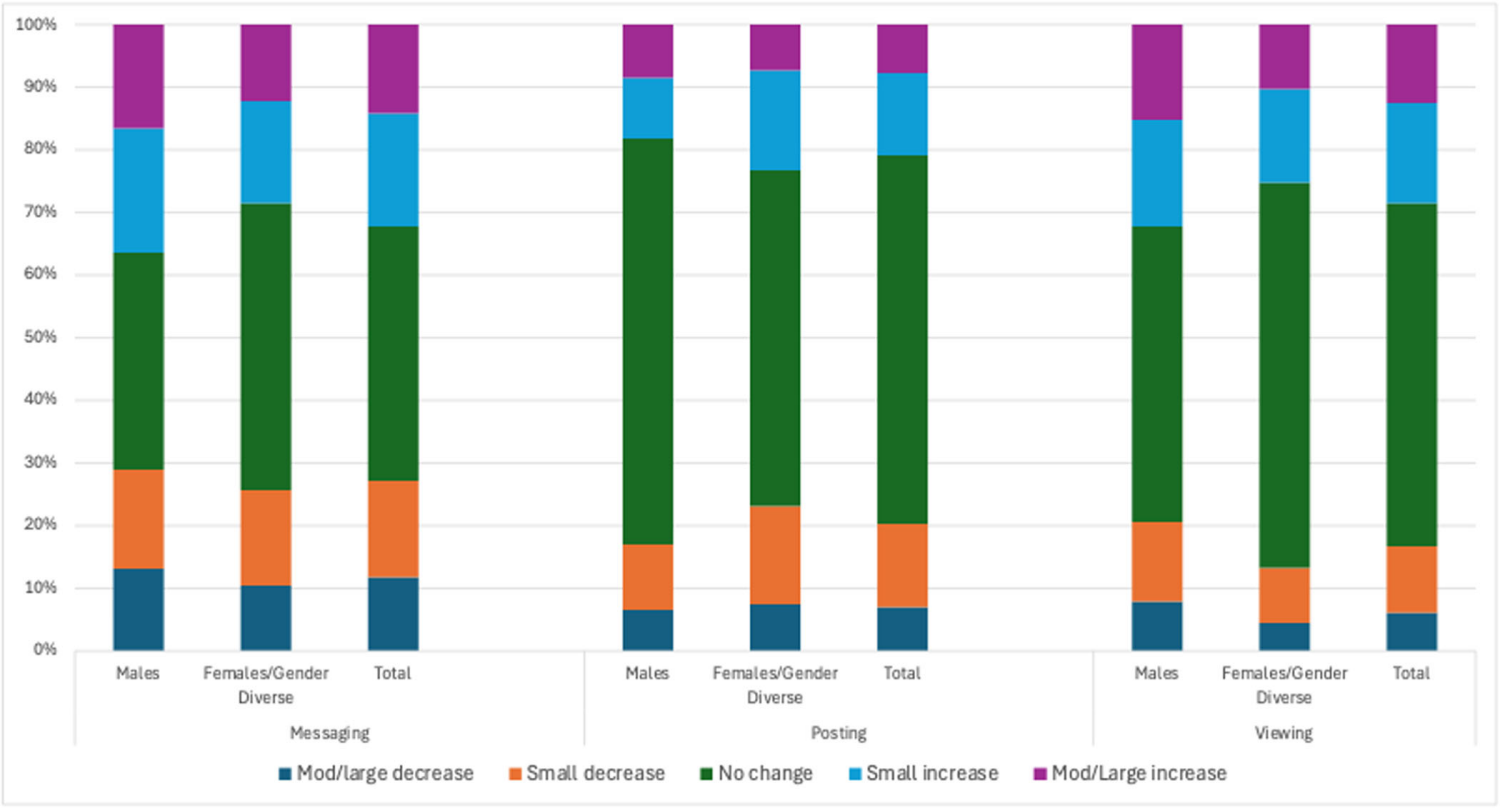
Change over time in each social media use behavior by gender.

The 12‐month change in the reported frequencies of the three social media behaviors is summarized in Figure 3. Overall, 40.7% of participants did not change their messaging/video calling frequency, 27.2% reported decreased frequency (15.5% small decrease, 11.7% moderate‐to‐large decrease), and 32.2% reported increased frequency (17.9% small increase, 14.3% moderate‐to‐large increase). More than half (58.8%) of participants did not change their posting frequency, 20.3% reported decreased frequency (13.3% small decrease, 7.0% moderate‐to‐large decrease), and 20.9% reported increased frequency (13.1% small increase, 7.8% moderate‐to‐large increase). More than half (54.9%) of participants reported no change in their viewing frequency, 16.6% reported decreased frequency (10.6% small decrease, 6.0% moderate‐to‐large decrease), and 28.5% reported increased frequency (15.9% small increase, 12.6% moderate‐to‐large increase).

As summarized in Figure 3 and Supporting Information S1: Table 3, male behaviors were less stable than female and gender diverse participants for messaging (no change reported by 34.7% males vs. 45.8% females and gender diverse, *p* < 0.001) and viewing content (no change reported by 47.1% of males vs. 61.6% of females and gender diverse, *p* < 0.001). However, male behaviors were more stable than females and gender diverse participants for posting content (no change reported by 64.8% of males vs. 53.6% of females and gender diverse, *p* < 0.001).

### 3.4 Associations Between Social Media Behaviors and Psychological Distress and Mental Wellbeing

Cross‐sectional, longitudinal prospective, and longitudinal change‐based associations between each social media behavior and psychological distress are summarized in Table 1 and full model output is available in Supporting Information S1: Tables 4–6. Cross‐sectionally at T2, a statistically significant relationship was observed between posting and high psychological distress, but only on weekdays (χ2 = 13.35, dr = 3, *p* = 0.0039). Compared to those who did not usually post on weekdays, posting every day or 3−4 days was associated with 70% and 50% higher odds of reporting high psychological distress, respectively (OR: 1.7, 95% CI: 1.1, 2.5 and OR: 1.5, 95% CI: 1.1, 2.0, respectively). There was some evidence of a relationship between change in posting over time and high psychological distress, however the *p*‐value associated with the chi‐squared statistic did not fall below 0.025. All other main effects indicated little evidence for cross‐sectional or longitudinal (prospective or change‐based) relationships between messaging or video calling friends, posting content or viewing content and high psychological distress.

**Table 1:** Associations between each social media behavior and psychological distress.

		Messaging (OR, 95% CI)	Posting (OR, 95% CI)	Viewing (OR, 95% CI)
*Cross‐sectional: T2 behavior ‐> T2 high psychological distress*				
Weekday	Every day	0.9 [0.7, 1.2]	1.7* [1.1, 2.5]	1.5 [1.0, 2.3]
	3−4 days	1.0 [0.7, 1.4]	1.5** [1.1, 2.0]	1.5 [0.9, 2.4]
	1−2 days	0.9 [0.6, 1.2]	1.2 [1.0, 1.6]	1.4 [0.9, 2.3]
	Not usually/never	1	1	1
		χ2 = 1.12 (df = 3), *p* = 0.7725	χ2 = 13.35 (df = 3), *p* = 0.0039	χ2 = 3.64 (df = 3), *p* = 0.3205
Weekend	Both days	1.0 [0.8, 1.3]	1.4 [1.0, 1.9]	1.2 [0.9, 1.8]
	One day	0.9 [0.6, 1.2]	1.1 [0.9, 1.4]	1.2 [0.9, 1.7]
	Not usually/not at all	1	1	1
		χ2 = 1.63 (df = 2), *p* = 0.4429	χ2 = 4.77 (df = 2), *p* = 0.0923	χ2 = 1.64 (df = 2), *p* = 0.4400
*Longitudinal: T1 behavior ‐> T2 high psychological distress*				
Weekday	Every day	0.9 [0.7, 1.1]	1.0 [0.7, 1.4]	1.0 [0.8, 1.4]
	3−4 days	1.0 [0.8, 1.3]	1.0 [0.7, 1.4]	0.9 [0.6, 1.3]
	1−2 days	1.1 [0.8, 1.4]	1.2 [0.9, 1.5]	1.2 [0.8, 1.8]
	Not usually/never	1	1	1
		χ2 = 2.88 (df = 3), *p* = 0.4109	χ2 = 2.59 (df = 3), *p* = 0.4594	χ2 = 2.51 (df = 3), *p* = 0.4731
Weekend	Both days	0.8 [0.7, 1.0]	1.0 [0.7, 1.4]	1.2 [0.9, 1.6]
	One day	0.8 [0.6, 1.1]	1.1 [0.9, 1.4]	1.4 [1.0, 2.0]
	Not usually/not at all	1	1	1
		χ2 = 2.97 (df = 2), *p* = 0.2263	χ2 = 0.81 (df = 2), *p* = 0.6659	χ2 = 2.92 (df = 2), *p* = 0.2325
*Changed‐based longitudinal: ΔT1:T2 (change in behavior) ‐> T2 (high psychological distress)*				
Weekly	Large decrease	0.9 [0.7, 1.1]	0.9 [0.6, 1.4]	0.9 [0.6, 1.4]
	Small decrease	0.9 [0.7, 1.1]	1.2 [0.9, 1.5]	1.1 [0.8, 1.4]
	No change	1	1.0	1.0
	Small increase	1.0 [0.8, 1.3]	1.2 [0.9, 1.6]	1.1 [0.8, 1.4]
	Large increase	1.1 [0.8, 1.3]	1.5* [1.1, 2.0]	1.3 [1.0, 1.6]
		χ2 = 4.02 (df = 4), *p* = 0.4027	χ2 = 10.55 (df = 4), *p* = 0.0320	χ2 = 3.98 (df = 4), *p* = 0.4088

*Note:* ***p < 0.001, **p < 0.01, *p < 0.025. OR = Odds Ratio, 95% CI = 95% Confidence Interval. Each behavior was modeled separately and models adjusted for gender, relative family affluence, intervention group, participant state, T2 screen time. Longitudinal and change‐based longitudinal models additionally adjusted for the relevant outcome at T1.

Associations between each social media behavior and mental wellbeing are summarized in Table 2 and full model output is available in Supporting Information S1: Tables 7–9. Cross‐sectionally at T2, associations were observed for messaging or video calling friends and high wellbeing, on both weekdays (χ2 = 10.16, df = 3, *p* = 0.0173) and weekends (χ2 = 8.06, df = 2, *p* = 0.0177). Compared to not usually messaging or video calling friends on weekdays, doing so every day or 1−2 days was associated with 40% and 50% higher odds of reporting high wellbeing, respectively (OR: 1.4, 95% CI: 1.1, 1.8 and OR: 1.5, 95% CI: 1.1, 2.1, respectively). Compared to not usually messaging or video calling friends on weekends, doing so both days or 1 day were both associated with 40% higher odds of reporting high wellbeing, respectively (OR: 1.4, 95% CI: 1.1, 1.9 and OR: 1.4, 95% CI: 1.0, 1.8, respectively). Main effects for all other models indicated little evidence for cross‐sectional or longitudinal (prospective or change‐based) relationships between posting or viewing content and high mental wellbeing.

**Table 2:** Associations between each social media behavior and mental wellbeing.

		Messaging (OR, 95% CI)	Posting (OR, 95% CI)	Viewing (OR, 95% CI)
*Cross‐sectional: T2 behavior ‐> T2 high mental wellbeing*				
Weekday	Every day	1.4** [1.1, 1.8]	1.0 [0.6, 1.6]	0.8 [0.6, 1.1]
	3–4 days	1.2 [0.9, 1.7]	0.7* [0.5, 0.9]	0.7 [0.5, 1.0]
	1–2 days	1.5* [1.1, 2.1]	1.0 [0.8, 1.3]	1.0 [0.7, 1.5]
	Not usually/never	1	1	1
		χ2 = 10.16 (df = 3), *p* = 0.0173	χ2 = 8.10 (df = 3), *p* = 0.0441	χ2 = 6.73 (df = 3), *p* = 0.0810
Weekend	Both days	1.4** [1.1, 1.9]	1.0 [0.8, 1.2]	0.8 [0.6, 1.0]
	One day	1.4* [1.0, 1.8]	1.1 [0.8, 1.4]	0.9 [0.7, 1.2]
	Not usually/not at all	1	1	1
		χ2 = 8.06 (df = 2), *p* = 0.0177	χ2 = 0.43 (df = 2), *p* = 0.8080	χ2 = 4.47 (df = 2), *p* = 0.1072
*Longitudinal: T1 behavior ‐> T2 high mental wellbeing*				
Weekday	Every day	1.1 [0.8, 1.4]	1.1 [0.8, 1.6]	1.0 [0.8, 1.2]
	3−4 days	0.8 [0.6, 1.1]	1.0 [0.7, 1.4]	1.2 [0.9, 1.7]
	1−2 days	1.2 [0.9, 1.6]	1.1 [0.9, 1.3]	0.9 [0.6, 1.3]
	Not usually/never	1	1	1
		χ2 = 6.05 (df = 3), *p* = 0.1092	χ2 = 0.68 (df = 3), *p* = 0.8783	χ2 = 5.78 (df = 3), *p* = 0.1230
Weekend	Both days	1.1 [0.9, 1.4]	1.3 [1.0, 1.8]	1.0 [0.8, 1.3]
	One day	1.1 [0.8, 1.4]	1.0 [0.8, 1.3]	1.0 [0.7, 1.4]
	Not usually/not at all	1	1	1
		χ2 = 0.78 (df = 2), *p* = 0.6772	χ2 = 3.62 (df = 2), *p* = 0.1633	χ2 = 0.04 (df = 2), *p* = 0.9825
*Changed‐based longitudinal: ΔT1:T2 (change in behavior) ‐> T2 (high mental wellbeing)*				
Weekly	Large decrease	0.9 [0.7, 1.2]	1.1 [0.8, 1.5]	1.2 [0.8, 1.7]
	Small decrease	1.1 [0.8, 1.4]	1.2 [0.9, 1.5]	1.1 [0.8, 1.5]
	No change	1	1.0	1.0
	Small increase	1.2 [0.9, 1.5]	1.0 [0.7, 1.3]	1.2 [0.9, 1.5]
	Large increase	1.2 [0.9, 1.5]	1.0 [0.7, 1.4]	0.9 [0.7, 1.1]
		χ2 = 4.06 (df = 4), *p* = 0.3983	χ2 = 2.02 (df = 4), *p* = 0.7327	χ2 = 4.83 (df = 4), *p* = 0.3057

*Note:* ***p < 0.001, **p < 0.01, *p < 0.025. OR = Odds Ratio, 95% CI = 95% Confidence Interval. Each behavior was modeled separately and models adjusted for gender, relative family affluence, intervention group, participant state, T2 screen time. Longitudinal and change‐based longitudinal models additionally adjusted for the relevant outcome at T1.

### 3.5 Interaction Effects and Subgroup Analyzes by Gender

Interaction analyzes did not identify any significant interactions for gender. As such, subgroup analysis was not conducted.

## 4 Discussion

This study employs a large, longitudinal adolescent data set to investigate the relationship between distinct social media behaviors and psychological distress and mental wellbeing. By utilizing a staged cross‐sectional then longitudinal analytic framework and examining *change* in three distinct social media behaviors, it extends current literature testing the active/passive model of social media behavior, which posits that passive use negatively impacts wellbeing and active use leads to beneficial effects on wellbeing (Valkenburg et al. 2021). Most existing studies testing this model are cross‐sectional, however there are two recently published longitudinal studies that examine the longitudinal relationship between active/passive social media behaviors and internalising symptoms (Tibbs et al. 2025) and life satisfaction (Boer et al. 2022), to which we compare our findings below. However, the present study complements these studies by examining both negative outcomes (psychological distress) and positive outcomes (mental wellbeing). This study also extends current literature by differentiating messaging or video calling friends from posting content, recognizing that—while both are “active” behaviors—the former is often more social and interactive than the latter, which may correspond to different associations with mental health and wellbeing.

Overall, analysis demonstrated very little evidence of a relationship between any of the three active or passive social media behaviors and psychological distress and mental wellbeing. There was some evidence of cross‐sectional associations between posting on social media and high psychological distress, and messaging or video calling friends and high wellbeing, but little to no evidence of any longitudinal associations for any of the three distinct social media behaviors and either psychological distress or mental wellbeing. Existing studies that have demonstrated support for the active/passive model of social media behavior and mental health and wellbeing have been cross‐sectional in nature (Verduyn et al. 2017; Chen et al. 2019; Thorisdottir et al. 2019; Kim et al. 2020; Verduyn et al. 2021). The two longitudinal studies from Tibbs et al and Boer et al also found little evidence for a relationship between active or passive use and mental health and wellbeing outcomes (internalizing symptoms and life satisfaction, respectively) over time (Boer et al. 2022; Tibbs et al. 2025). Taken together with the results of the present study, these findings suggest there is little evidence to date of a relationship between active or passive social media behavior and either psychological distress or wellbeing.

Some existing cross‐sectional studies suggest that relationships between active/passive social media behavior and mental health differ by gender (Thorisdottir et al. 2019; Svensson et al. 2022). Whilst the present study did find differences in the frequency and change over time in each of the social media behaviors by gender, we found no evidence of any interaction effect for gender when modeling the longitudinal relationship between change in any of the behaviors and psychological distress or mental wellbeing. This was consistent with the longitudinal analysis from Tibbs et al. 2025, highlighting little support for a link between gender‐based differences in social media behavior and gender‐based differences in mental health.

### 4.1 Strengths and Limitations

This study has several notable strengths. First, it uses a large, contemporary general community sample across three geographically diverse Australian states. Second, it uses a comprehensive analytic strategy with cross‐sectional analysis for comparability with existing literature and then both prospective and change‐based longitudinal analysis, spanning a developmentally important timeframe capturing the last 2 years of mandatory schooling in Australia (age 14−16). The change‐based analysis is particularly unique as it quantifies the impact of within‐person change in social media behaviors on mental health and wellbeing. Third, models account for important sociodemographic confounders and adjust for screen time duration, allowing investigation of the role of the distinct social media behaviors, over‐and‐above the effects of general time spent. Fourth, it actively examines gender interactions, rather than simply accounting for gender as a covariate. Fifth, it extends current treatment of active and passive behavior in extant literature—which generally groups all active and all passive behaviors—by differentiating more social and interactive (messaging and video calling friends) active behavior from less social and interactive (posting content) active behavior. It also differentiates weekend and weekday behaviors, recognizing that for school‐aged adolescents there are likely to be differences between the two, due to different time use and the potential for different parent rules (Sigmundová and Sigmund 2021; Reardon et al. 2023). Finally, it examines associations with both harms and benefits by including both psychological distress and mental wellbeing as outcomes.

Despite these strengths, findings should be interpreted with consideration of several limitations. First, the active and passive social media measure was developed in‐house due to the lack of appropriate measures available at the time. While it was focus‐tested with several adolescents, it did not undergo rigorous psychometric testing. Second, all measures were self‐report and thus might be subject to recall bias or social desirability bias (although participants were assured of the confidentiality of their responses to mitigate this). This is particularly important given the observed modest agreement between self‐report and more objective measures of social media use, that is, actual total screen time logged by the phone itself (Steele et al. 2023) and the finding that discrepancies between subjective and actual social media use may be mediated by the degree of psychosocial impairment (Sewall et al. 2022). Some have even called for researchers to abandon subjective measures altogether (Molaib et al. 2025). Acknowledging the challenges associated with objective data collection, future research will benefit from multimodal assessment of social media use, incorporating both self‐report and objective measurement. Third, the sample was not population representative as it contained more schools with higher socio‐educational advantage and more schools in urban areas compared to all Australian schools. Fourth, psychological distress and mental wellbeing are potentially influenced by many individual, peer, family, community and broader societal factors (Halladay et al. 2024; Smout et al. 2025) which were not measured in the current study. Finally, results may have been influenced by the COVID‐19 pandemic as data at timepoint one were collected in late 2021, when there were still some restrictions in place in Australia. We cannot rule out that these restrictions impacted social media behaviors, psychological distress, and wellbeing.

### 4.2 Future Directions for Research and Policy

Findings from this study suggest the need to move beyond the active and passive model of social media behavior as a framework to examine and explain the relationship between social media and adolescent mental health. Moreover, future research should prioritize direct measurement of online behaviors (e.g., number of posts, likes, time‐stamped activity logs) and the validation of self‐report scales against objective data to improve the precision of social media use assessments among adolescents. Several recently published studies offer compelling new frameworks and measurement tools through which to consider this relationship. A comprehensive review of the mechanisms through which social media may impact adolescent mental health makes the case for considering the affordances of social media (e.g., anonymity, availability, editability, and quantifiability) and the way in which these interact with unique behavioral, cognitive, and neurobiological vulnerabilities of adolescent development to impact mental health (Orben et al. 2024). This review highlights gaps in extant literature for well‐designed longitudinal studies that investigate these mechanisms using diverse methods (such as survey data, neuroimaging, digital trace and other objectively measured data). Further, three conceptualizations and measurement tools that move beyond the active passive model have been proposed in other recent studies. First, Tuck and Thompson propose the Social Media Use Scale, which conducts a rigorous development and psychometric evaluation process, initially testing an extension of the active/passive model, but culminating in a measure that moves away from this, instead differentiating belief‐based, consumption‐based, image‐based, and comparison‐based social media (Tuck and Thompson 2023). The resulting Social Media Use Scale offers a promising measure to investigate some of the mechanistic knowledge gaps identified in the aforementioned review (Orben et al. 2024; Tuck and Thompson 2023). Two other conceptualizations of social media behavior have emerged as promising new directions through which to investigate the relationship between social media and mental health; “digital stress” and “digital flourishing” (Orben et al. 2024; Nick et al. 2022; Rosič et al. 2022). Digital stress has many definitions but includes stressors such as cyberbullying, pressure to be available, fear of missing out, and communication overload (Orben et al. 2024). More research is needed to develop and psychometrically evaluate a scale to measure digital stress, however a brief scale that was adapted by Nick, Zilic et. al. found evidence that digital stress was longitudinally associated with depressive symptoms among adolescents (Nick et al. 2022). Conversely, digital flourishing is defined as self‐perceived positive experiences and behaviors in digital communication, across the domains of connectedness, positive social comparison, authentic self‐presentation, civil participation, and self‐control (Rosič et al. 2022).

Findings from the present study and other reviews have demonstrated that the relationship between social media behaviors and mental health is nuanced (with both harms and benefits) and highly individual (Orben et al. 2024; Valkenburg et al. 2022). This raises concerns about the moves from governments around the world to impose blanket bans on access to social media among adolescents. In Australia, where the present study was based, the Government recently announced a social media ban for all adolescents under 16 years. The European Union tried to introduce a similar ban in 2015 (Henley 2015), but eventually it was determined that individual member countries could select and enforce their own limits. Since then, France has introduced a law that requires parental consent for teens under 15 to access social media (Observatory 2023) and other European countries have adopted various other approaches. Korea attempted to introduce time restrictions for adolescent online gaming, but these were easily thwarted through the use of adults' accounts (Kattula et al. 2021). China has legislated age‐based time and content restrictions that restrict access to all content except educational materials for young children and heavily censor the tone and themes of content for older children (Translate n.d). Notwithstanding issues with enforcement that have been faced by nearly all jurisdictions that have attempted age‐based bans, there is a lack of evidence that bans will offer any mental health benefits. Further, there are concerns that they may reduce safety online for adolescents who will resort to secret use, as parental oversight and monitoring are key strategies to enhance online safety (Nagata et al. 2025), and may isolate marginalized adolescents who rely on social media‐based communication for social connection (Karim et al. 2022; Craig et al. 2021). While further evidence on the mechanisms and intervention targets emerges, it would be prudent for policymakers to focus investment on education, to engage in meaningful, well‐designed consultation with young people, and to rigorously evaluate the impact of social media restrictions on youth mental health (Christensen et al. 2024).

## Ethics Statement

The Health4Life trial had ethical approval from the Human Research Ethics Committees of the University of Sydney (2018/882), Curtin University (HRE2019‐0083), the University of Queensland (2019000037), and relevant school sector ethics committees.

## Conflicts of Interest

The authors declare no conflicts of interest.

## Supporting information

JAD‐2025‐0083 supplementary materials R1.

## Data Availability

De‐identified participant data will be made available to researchers on reasonable request to K.E.C. (katrina.champion@sydney.edu.au) when accompanied by study protocol and analysis plan. Data will be shared after the approval of a proposal by a committee of the current research team with a signed data access agreement.
