# Supplementary material for: Scrolling, Chatting, and Posting: Longitudinal Changes in Distinct Social Media Behaviors and Their Relationship With Psychological Distress and Mental Wellbeing in Adolescents
Source: J Adolesc. 2025 Sep 24;98(1):237–49. doi: 10.1002/jad.70055 (PMC12780657; doi:10.1002/jad.70055)
Supplement: Supplementary file 1 — JAD‐2025‐0083 supplementary materials R1. [file JAD-98-237-s001.docx]

# Supplementary materials

**Supplementary note 1.**

While not available at the time of data collection for the surrent study, subsequent measures of active and passive social media use have been developed and at least partially validated (for example, the Social Media Activity Questionnaire. Following is a short description of potential measures investigated at the time of questionnaire development in late 2020-early 2021.

In Coyne et al. 2021, participants reported how much time they spent on a typical day watching TV programs (on any device), playing video games (online or offline), talking on a cell phone, texting on a cell phone, and using social networking sites (e.g., Facebook, Instagram, and Twitter). They responded on a 9-point Likert scale (1 = none, 2 = Less than 20 min, 3 = 31–60 minutes, 4 = 1–2 h, 5 = 2–3 h, 6 = 3–4 h, 7 = 5–6 h, 8 = 7–8 h, 9 = More than 8 hours) [1].

In two studies led by Boers in 2019 [2] and 2020 [3] screen time was measured by how much time per day one spends playing video games (on a computer, cell phone, game console), using social media (Facebook, Twitter, or other social networking sites), watching shows or movies (on television or the computer), and on the computer engaging in other activities. The amount of screen time was operationalized into four categories: 0 to 30 minutes, 30 minutes to 1 hour 30 minutes, 1 hour 30 minutes to 2 hour 30 minutes, and 3 hour 30 minutes or more.

In Kaur et al. 2020, students were asked “About how many hours on an average day do you spend: a. playing games on a computer, TV, phone, or other electronic device? b. texting? c. talking on the phone? d. on social networking Web sites like Facebook, Twitter, Instagram, etc.? e. video chatting (Skype, etc.)?” Scales were: none, less than 1 h, 1−2 hours, 3−4 hours, 5−6 hours, 7−8 hours, and 9 hours or more [4].

In Beyens et al. 2020, each participant was asked to report their use of three social media platforms that they used most frequently out of WhatsApp, Instagram, Snapchat, YouTube, and, the chat function of games. The questions distinguished between active and passive social media use. For example, questions for Instagram were: 1) “How much time did you spend in the last hour on viewing posts/stories of others on Instagram?” (passive), 2) “How much time did you spend in the last hour on posting on your feed, or sharing a story on Instagram?” (active 1), and 3) “How much time did you spend in the last hour on sending direct messages/chatting on Instagram?” [5].

**Supplementary Table 1. Screen behaviour measure developed for the Health4Life study**

| In a typical school week (Monday to Friday), how many days out of five do you… | Every day | 3-4 days | 1-2 days | Less than once per school week | Not at all |
| --- | --- | --- | --- | --- | --- |
| 1. Message or video call friends   (on platforms such as Snapchat, Instagram, FaceTime, Whatsapp, text message etc) |  |  |  |  |  |
| 1. **Post content on social media**  (stories, feed posts, videos etc) |  |  |  |  |  |
| 1. **View other peoples’ content on social media**  (watching videos, scrolling through posts and stories etc) |  |  |  |  |  |

| In a typical weekend (Saturday and Sunday), how many days do you… | Both days | One day | Less than once per weekend | Not at all |
| --- | --- | --- | --- | --- |
| 1. Message or video call friends   (on platforms such as Snapchat, Instagram, FaceTime, Whatsapp, text message etc) |  |  |  |  |
| 1. **Post content on social media**  (stories, feed posts, videos etc) |  |  |  |  |
| 1. **View other peoples’ content on social media**  (watching videos, scrolling through posts and stories etc) |  |  |  |  |

**Supplementary Note 2. Recoding to combine weekday and weekend and calculate change over time**

In order to calculate change over time in social media behaviours, weekday and weekend responses were combined as below:

| Weekday answer | Weekend answer | Recoded to |
| --- | --- | --- |
| Every day | Both days | 7 days |
| 3-4 days | Both days | 5.5 days |
| 1-2 days | Both days | 3.5 days |
| Not usually / Not at all | Both days | 2 days |
| Every day | One day | 6 days |
| 3-4 days | One day | 4.5 days |
| 1-2 days | One day | 2.5 days |
| Not usually / Not at all | One day | 1 day |
| Every day | Not usually / Not at all | 5 days |
| 3-4 days | Not usually / Not at all | 3.5 days |
| 1-2 days | Not usually / Not at all | 1.5 days |
| Not usually / Not at all | Not usually / Not at all | Not usually / Not at all |

Change between timepoint 1 and 2 was then calculated from this recoded variable, and categorised as: large decrease (a decrease in frequency of 3 or more days a week); small decrease (a decrease of less than 3 but more than 0 days a week); no change; small increase (an increase of more than 0 but less than 3 days a week); and large increase (an increase of 3 or more days a week).

**Supplementary Table 2. Prevalence and frequency of each social media use behaviour by timepoint and gender**

|  | Male (N=1,487) | Female & gender diverse (N=1,718) | Total (N=3,205) | p-value |
| --- | --- | --- | --- | --- |
| Weekday messaging freq at T1 |  |  |  |  |
| Every day | 723 (48.6%) | 1,060 (61.7%) | 1,783 (55.6%) | <0.001 |
| 3-4 days | 272 (18.3%) | 278 (16.2%) | 550 (17.2%) |  |
| 1-2 days | 224 (15.1%) | 233 (13.6%) | 457 (14.3%) |  |
| Not usually / Never | 268 (18.0%) | 147 (8.6%) | 415 (12.9%) |  |
| Weekday messaging freq at T2 |  |  |  |  |
| Every day | 790 (53.1%) | 1,114 (64.8%) | 1,904 (59.4%) | <0.001 |
| 3-4 days | 243 (16.3%) | 250 (14.6%) | 493 (15.4%) |  |
| 1-2 days | 247 (16.6%) | 223 (13.0%) | 470 (14.7%) |  |
| Not usually / Never | 207 (13.9%) | 131 (7.6%) | 338 (10.5%) |  |
| Weekend messaging freq at T1 |  |  |  |  |
| Both days | 841 (56.6%) | 1,155 (67.2%) | 1,996 (62.3%) | <0.001 |
| One day | 341 (22.9%) | 369 (21.5%) | 710 (22.2%) |  |
| Not usually / Not at all | 305 (20.5%) | 194 (11.3%) | 499 (15.6%) |  |
| Weekend messaging freq at T2 |  |  |  |  |
| Both days | 931 (62.6%) | 1,202 (70.0%) | 2,133 (66.6%) | <0.001 |
| One day | 316 (21.3%) | 340 (19.8%) | 656 (20.5%) |  |
| Not usually / Not at all | 240 (16.1%) | 176 (10.2%) | 416 (13.0%) |  |
| Weekday posting freq at T1 |  |  |  |  |
| Every day | 80 (5.4%) | 124 (7.2%) | 204 (6.4%) | <0.001 |
| 3-4 days | 69 (4.6%) | 137 (8.0%) | 206 (6.4%) |  |
| 1-2 days | 171 (11.5%) | 349 (20.3%) | 520 (16.2%) |  |
| Not usually / Never | 1,167 (78.5%) | 1,108 (64.5%) | 2,275 (71.0%) |  |
| Weekday posting freq at T2 |  |  |  |  |
| Every day | 84 (5.6%) | 93 (5.4%) | 177 (5.5%) | <0.001 |
| 3-4 days | 78 (5.2%) | 154 (9.0%) | 232 (7.2%) |  |
| 1-2 days | 171 (11.5%) | 370 (21.5%) | 541 (16.9%) |  |
| Not usually / Never | 1,154 (77.6%) | 1,101 (64.1%) | 2,255 (70.4%) |  |
| Weekend posting freq at T1 |  |  |  |  |
| Both days | 91 (6.1%) | 174 (10.1%) | 265 (8.3%) | <0.001 |
| One day | 186 (12.5%) | 406 (23.6%) | 592 (18.5%) |  |
| Not usually / Not at all | 1,210 (81.4%) | 1,138 (66.2%) | 2,348 (73.3%) |  |
| Weekend posting freq at T2 |  |  |  |  |
| Both days | 117 (7.9%) | 177 (10.3%) | 294 (9.2%) | <0.001 |
| One day | 202 (13.6%) | 430 (25.0%) | 632 (19.7%) |  |
| Not usually / Not at all | 1,168 (78.5%) | 1,111 (64.7%) | 2,279 (71.1%) |  |
| Weekday viewing freq at T1 |  |  |  |  |
| Every day | 798 (53.7%) | 1,187 (69.1%) | 1,985 (61.9%) | <0.001 |
| 3-4 days | 266 (17.9%) | 237 (13.8%) | 503 (15.7%) |  |
| 1-2 days | 187 (12.6%) | 162 (9.4%) | 349 (10.9%) |  |
| Not usually / Never | 236 (15.9%) | 132 (7.7%) | 368 (11.5%) |  |
| Weekday viewing freq at T2 |  |  |  |  |
| Every day | 929 (62.5%) | 1,316 (76.6%) | 2,245 (70.0%) | <0.001 |
| 3-4 days | 232 (15.6%) | 203 (11.8%) | 435 (13.6%) |  |
| 1-2 days | 165 (11.1%) | 121 (7.0%) | 286 (8.9%) |  |
| Not usually / Never | 161 (10.8%) | 78 (4.5%) | 239 (7.5%) |  |
| Weekend viewing freq at T1 |  |  |  |  |
| Both days | 921 (61.9%) | 1,302 (75.8%) | 2,223 (69.4%) | <0.001 |
| One day | 284 (19.1%) | 237 (13.8%) | 521 (16.3%) |  |
| Not usually / Not at all | 282 (19.0%) | 179 (10.4%) | 461 (14.4%) |  |
| Weekend viewing freq at T2 |  |  |  |  |
| Both days | 1,022 (68.7%) | 1,430 (83.2%) | 2,452 (76.5%) | <0.001 |
| One day | 259 (17.4%) | 193 (11.2%) | 452 (14.1%) |  |
| Not usually / Not at all | 206 (13.9%) | 95 (5.5%) | 301 (9.4%) |  |
| p-value is from Pearson chi-squared statistic testing gender differences | | | | |

**Supplementary Table 3. Twelve-month change in the frequency of each social media use behaviour by gender**

|  | Male (N=1,487) | Female (N=1,718) | Total (N=3,205) | p-value |
| --- | --- | --- | --- | --- |
| Change in messaging between T1 and T2 |  |  |  |  |
| large decrease | 195 (13.1%) | 180 (10.5%) | 375 (11.7%) | <0.001 |
| small decrease | 235 (15.8%) | 261 (15.2%) | 496 (15.5%) |  |
| no change | 516 (34.7%) | 787 (45.8%) | 1,303 (40.7%) |  |
| small increase | 294 (19.8%) | 281 (16.4%) | 575 (17.9%) |  |
| large increase | 247 (16.6%) | 209 (12.2%) | 456 (14.2%) |  |
| Change in posting between T1 and T2 |  |  |  |  |
| large decrease | 97 (6.5%) | 128 (7.5%) | 225 (7.0%) | <0.001 |
| small decrease | 156 (10.5%) | 270 (15.7%) | 426 (13.3%) |  |
| no change | 964 (64.8%) | 921 (53.6%) | 1,885 (58.8%) |  |
| small increase | 145 (9.8%) | 274 (15.9%) | 419 (13.1%) |  |
| large increase | 125 (8.4%) | 125 (7.3%) | 250 (7.8%) |  |
| Change in viewing between T1 and T2 |  |  |  |  |
| large decrease | 118 (7.9%) | 75 (4.4%) | 193 (6.0%) | <0.001 |
| small decrease | 188 (12.6%) | 152 (8.8%) | 340 (10.6%) |  |
| no change | 701 (47.1%) | 1,058 (61.6%) | 1,759 (54.9%) |  |
| small increase | 253 (17.0%) | 257 (15.0%) | 510 (15.9%) |  |
| large increase | 227 (15.3%) | 176 (10.2%) | 403 (12.6%) |  |
| Female = female and gender diverse combined; p-value is from Pearson chi-squared statistic testing gender differences | | | | |

**Supplementary Table 4. Cross-sectional, prospective longitudinal, and longitudinal change-based models of the relationship between messaging/video calling friends and high psychological distress**

|  | Cross-sectional (T2) Weekday | Cross-sectional (T2) Weekend | Prospective longitudinal (T1🡪 T2) Weekday | Prospective longitudinal (T1🡪 T2) Weekend | Longitudinal change (between T1 & T2) |
| --- | --- | --- | --- | --- | --- |
| Main effect chi-square test | **X^2^(3)=1.12, p=0.773** | **X^2^(2)=1.63, p=0.443** | **X^2^(3)=2.88, p=0.411** | **X^2^(2)=2.97, p=0.226** | **X^2^(4)=4.02, p=0.403** |
| Gender |  |  |  |  |  |
| Male | 1.0 | 1.0 | 1.0 | 1.0 | 1.0 |
| Female | 2.7*** [2.3, 3.3] | 2.7*** [2.3, 3.3] | 2.1*** [1.7, 2.6] | 2.1*** [1.7, 2.6] | 2.1*** [1.7, 2.5] |
| SES |  |  |  |  |  |
| Lowest 20% | 1.0 | 1.0 | 1.0 | 1.0 | 1.0 |
| Middle 60% | 0.8 [0.6, 1.0] | 0.8 [0.6, 1.0] | 0.8 [0.6, 1.1] | 0.8 [0.6, 1.1] | 0.8 [0.6, 1.1] |
| Upper 20% | 0.8 [0.6, 1.0] | 0.8 [0.6, 1.0] | 0.8 [0.6, 1.1] | 0.8 [0.6, 1.1] | 0.8 [0.6, 1.1] |
| Trial group |  |  |  |  |  |
| Control | 1.0 | 1.0 | 1.0 | 1.0 | 1.0 |
| Intervention | 0.9 [0.7, 1.0] | 0.9 [0.7, 1.0] | 0.8 [0.7, 1.0] | 0.8 [0.7, 1.0] | 0.8 [0.7, 1.0] |
| State |  |  |  |  |  |
| NSW | 1.0 | 1.0 | 1.0 | 1.0 | 1.0 |
| QLD | 0.8 [0.6, 1.0] | 0.8 [0.6, 1.0] | 0.8 [0.7, 1.1] | 0.8 [0.7, 1.1] | 0.8 [0.7, 1.1] |
| WA | 0.9 [0.7, 1.2] | 0.9 [0.7, 1.2] | 1.0 [0.7, 1.3] | 1.0 [0.7, 1.3] | 1.0 [0.7, 1.3] |
| Average daily T2 screen time (hrs) | 1.1*** [1.1, 1.1] | 1.1*** [1.1, 1.1] | 1.1*** [1.1, 1.1] | 1.1*** [1.1, 1.1] | 1.1*** [1.1, 1.1] |
| Weekday messaging freq at T2 |  |  |  |  |  |
| Every day | 0.9 [0.7, 1.2] |  |  |  |  |
| 3-4 days | 1.0 [0.7, 1.4] |  |  |  |  |
| 1-2 days | 0.9 [0.6, 1.2] |  |  |  |  |
| Not usually / Never | 1.0 |  |  |  |  |
| Weekend messaging freq at T2 |  |  |  |  |  |
| Both days |  | 1.0 [0.8, 1.3] |  |  |  |
| One day |  | 0.9 [0.6, 1.2] |  |  |  |
| Not usually / Not at all |  | 1.0 |  |  |  |
| T1 High psychological distress |  |  |  |  |  |
| No |  |  | 1.0 | 1.0 | 1.0 |
| Yes |  |  | 6.8*** [5.4, 8.5] | 6.8*** [5.4, 8.5] | 6.8*** [5.4, 8.5] |
| Weekday messaging freq at T1 |  |  |  |  |  |
| Every day |  |  | 0.9 [0.7, 1.1] |  |  |
| 3-4 days |  |  | 1.0 [0.8, 1.3] |  |  |
| 1-2 days |  |  | 1.1 [0.8, 1.4] |  |  |
| Not usually / Never |  |  | 1.0 |  |  |
| Weekend messaging freq at T1 |  |  |  |  |  |
| Both days |  |  |  | 0.8 [0.7, 1.0] |  |
| One day |  |  |  | 0.8 [0.6, 1.1] |  |
| Not usually / Not at all |  |  |  | 1.0 |  |
| Change in messaging between T1 and T2 |  |  |  |  |  |
| large decrease |  |  |  |  | 0.9 [0.7, 1.1] |
| small decrease |  |  |  |  | 0.9 [0.7, 1.1] |
| no change |  |  |  |  | 1.0 |
| small increase |  |  |  |  | 1.0 [0.8, 1.3] |
| large increase |  |  |  |  | 1.1 [0.8, 1.3] |
| Intercept | 0.1*** [0.1, 0.2] | 0.1*** [0.1, 0.2] | 0.1*** [0.1, 0.2] | 0.1*** [0.1, 0.2] | 0.1*** [0.1, 0.1] |
| *** p<.001, ** p<.01, * p<.025 | | | | | |

**Supplementary Table 5. Cross-sectional, prospective longitudinal, and longitudinal change-based models of the relationship between posting and high psychological distress**

|  | Cross-sectional (T2) Weekday | Cross-sectional (T2) Weekend | Prospective longitudinal (T1🡪 T2) Weekday | Prospective longitudinal (T1🡪 T2) Weekend | Longitudinal change (between T1 & T2) |
| --- | --- | --- | --- | --- | --- |
| Main effect chi-square test | **X^2^(3)=13.35, p=0.004** | **X^2^(2)=4.77, p=0.092** | **X^2^(3)=2.59, p=0.459** | **X^2^(2)=0.81, p=0.666** | **X^2^(4)=10.55, p=0.032** |
| Gender |  |  |  |  |  |
| Male | 1.0 | 1.0 | 1.0 | 1.0 | 1.0 |
| Female | 2.6*** [2.2, 3.2] | 2.7*** [2.2, 3.3] | 2.0*** [1.6, 2.5] | 2.0*** [1.6, 2.5] | 2.0*** [1.6, 2.5] |
| SES |  |  |  |  |  |
| Lowest 20% | 1.0 | 1.0 | 1.0 | 1.0 | 1.0 |
| Middle 60% | 0.8 [0.6, 1.0] | 0.8 [0.6, 1.0] | 0.8 [0.6, 1.1] | 0.8 [0.6, 1.1] | 0.8 [0.6, 1.1] |
| Upper 20% | 0.8 [0.6, 1.0] | 0.8 [0.6, 1.0] | 0.8 [0.6, 1.1] | 0.8 [0.6, 1.1] | 0.8 [0.6, 1.1] |
| Trial group |  |  |  |  |  |
| Control | 1.0 | 1.0 | 1.0 | 1.0 | 1.0 |
| Intervention | 0.9 [0.7, 1.0] | 0.9 [0.7, 1.0] | 0.8 [0.7, 1.0] | 0.8 [0.7, 1.0] | 0.8 [0.7, 1.0] |
| State |  |  |  |  |  |
| NSW | 1.0 | 1.0 | 1.0 | 1.0 | 1.0 |
| QLD | 0.8 [0.7, 1.0] | 0.8 [0.7, 1.0] | 0.8 [0.7, 1.1] | 0.8 [0.7, 1.1] | 0.9 [0.7, 1.1] |
| WA | 1.0 [0.8, 1.2] | 1.0 [0.7, 1.2] | 1.0 [0.7, 1.3] | 1.0 [0.7, 1.3] | 1.0 [0.7, 1.3] |
| Average daily T2 screen time (hrs) | 1.1*** [1.1, 1.1] | 1.1*** [1.1, 1.1] | 1.1*** [1.1, 1.1] | 1.1*** [1.1, 1.1] | 1.1*** [1.0, 1.1] |
| Weekday posting freq at T2 |  |  |  |  |  |
| Every day | 1.7* [1.1, 2.5] |  |  |  |  |
| 3-4 days | 1.5** [1.1, 2.0] |  |  |  |  |
| 1-2 days | 1.2 [1.0, 1.6] |  |  |  |  |
| Not usually / Never | 1.0 |  |  |  |  |
| Weekend posting freq at T2 |  |  |  |  |  |
| Both days |  | 1.4 [1.0, 1.9] |  |  |  |
| One day |  | 1.1 [0.9, 1.4] |  |  |  |
| Not usually / Not at all |  | 1.0 |  |  |  |
| T1 High psych distress |  |  |  |  |  |
| No |  |  | 1.0 | 1.0 | 1.0 |
| Yes |  |  | 6.8*** [5.4, 8.5] | 6.8*** [5.4, 8.5] | 6.8*** [5.4, 8.5] |
| Weekday posting freq at T1 |  |  |  |  |  |
| Every day |  |  | 1.0 [0.7, 1.4] |  |  |
| 3-4 days |  |  | 1.0 [0.7, 1.4] |  |  |
| 1-2 days |  |  | 1.2 [0.9, 1.5] |  |  |
| Not usually / Never |  |  | 1.0 |  |  |
| Weekend posting freq at T1 |  |  |  |  |  |
| Both days |  |  |  | 1.0 [0.7, 1.4] |  |
| One day |  |  |  | 1.1 [0.9, 1.4] |  |
| Not usually / Not at all |  |  |  | 1.0 |  |
| Change in posting between T1 and T2 |  |  |  |  |  |
| large decrease |  |  |  |  | 0.9 [0.6, 1.4] |
| small decrease |  |  |  |  | 1.2 [0.9, 1.5] |
| no change |  |  |  |  | 1.0 |
| small increase |  |  |  |  | 1.2 [0.9, 1.6] |
| large increase |  |  |  |  | 1.5* [1.1, 2.0] |
| Intercept | 0.1*** [0.1, 0.2] | 0.1*** [0.1, 0.2] | 0.1*** [0.1, 0.1] | 0.1*** [0.1, 0.1] | 0.1*** [0.1, 0.1] |
| *** p<.001, ** p<.01, * p<.025 | | | | | |

**Supplementary Table 6. Cross-sectional, prospective longitudinal, and longitudinal change-based models of the relationship between viewing content and high psychological distress**

|  | Cross-sectional (T2) Weekday | Cross-sectional (T2) Weekend | Prospective longitudinal (T1🡪 T2) Weekday | Prospective longitudinal (T1🡪 T2) Weekend | Longitudinal change (between T1 & T2) |
| --- | --- | --- | --- | --- | --- |
| Main effect chi-square test | **X^2^(3)=3.64, p=0.303** | **X^2^(2)=1.64, p=0.440** | **X^2^(3)=2.51, p=0.473** | **X^2^(2)=2.92, p=0.233** | **X^2^(4)=3.98, p=0.409** |
| Gender |  |  |  |  |  |
| Male | 1.0 | 1.0 | 1.0 | 1.0 | 1.0 |
| Female | 2.7*** [2.2, 3.3] | 2.7*** [2.2, 3.3] | 2.1*** [1.7, 2.6] | 2.0*** [1.7, 2.5] | 2.1*** [1.7, 2.6] |
| SES |  |  |  |  |  |
| Lowest 20% | 1.0 | 1.0 | 1.0 | 1.0 | 1.0 |
| Middle 60% | 0.8 [0.6, 1.0] | 0.8 [0.6, 1.0] | 0.8 [0.6, 1.1] | 0.8 [0.6, 1.1] | 0.8 [0.6, 1.1] |
| Upper 20% | 0.8 [0.6, 1.0] | 0.8 [0.6, 1.0] | 0.8 [0.6, 1.1] | 0.8 [0.6, 1.1] | 0.8 [0.6, 1.1] |
| Trial group |  |  |  |  |  |
| Control | 1.0 | 1.0 | 1.0 | 1.0 | 1.0 |
| Intervention | 0.9 [0.7, 1.0] | 0.9 [0.7, 1.0] | 0.8 [0.7, 1.0] | 0.8 [0.7, 1.0] | 0.8 [0.7, 1.0] |
| State |  |  |  |  |  |
| NSW | 1.0 | 1.0 | 1.0 | 1.0 | 1.0 |
| QLD | 0.8 [0.6, 1.0] | 0.8 [0.6, 1.0] | 0.8 [0.7, 1.1] | 0.8 [0.7, 1.1] | 0.8 [0.7, 1.1] |
| WA | 0.9 [0.7, 1.2] | 0.9 [0.7, 1.2] | 1.0 [0.7, 1.3] | 1.0 [0.7, 1.3] | 1.0 [0.7, 1.3] |
| Average daily T2 screen time (hrs) | 1.1*** [1.1, 1.1] | 1.1*** [1.1, 1.1] | 1.1*** [1.1, 1.1] | 1.1*** [1.1, 1.1] | 1.1*** [1.1, 1.1] |
| Weekday viewing freq at T2 |  |  |  |  |  |
| Every day | 1.5 [1.0, 2.3] |  |  |  |  |
| 3-4 days | 1.5 [0.9, 2.4] |  |  |  |  |
| 1-2 days | 1.4 [0.9, 2.3] |  |  |  |  |
| Not usually / Never | 1.0 |  |  |  |  |
| Weekend viewing freq at T2 |  |  |  |  |  |
| Both days |  | 1.2 [0.9, 1.8] |  |  |  |
| One day |  | 1.2 [0.9, 1.7] |  |  |  |
| Not usually / Not at all |  | 1.0 |  |  |  |
| T1 High psych distress |  |  |  |  |  |
| No |  |  | 1.0 | 1.0 | 1.0 |
| Yes |  |  | 6.8*** [5.4, 8.5] | 6.8*** [5.4, 8.5] | 6.9*** [5.5, 8.6] |
| Weekday viewing freq at T1 |  |  |  |  |  |
| Every day |  |  | 1.0 [0.8, 1.4] |  |  |
| 3-4 days |  |  | 0.9 [0.6, 1.3] |  |  |
| 1-2 days |  |  | 1.2 [0.8, 1.8] |  |  |
| Not usually / Never |  |  | 1.0 |  |  |
| Weekend viewing freq at T1 |  |  |  |  |  |
| Both days |  |  |  | 1.2 [0.9, 1.6] |  |
| One day |  |  |  | 1.4 [1.0, 2.0] |  |
| Not usually / Not at all |  |  |  | 1.0 |  |
| Change in viewing between T1 and T2 |  |  |  |  |  |
| large decrease |  |  |  |  | 0.9 [0.6, 1.4] |
| small decrease |  |  |  |  | 1.1 [0.8, 1.4] |
| no change |  |  |  |  | 1.0 |
| small increase |  |  |  |  | 1.1 [0.8, 1.4] |
| large increase |  |  |  |  | 1.3 [1.0, 1.6] |
| Intercept | 0.1*** [0.1, 0.2] | 0.1*** [0.1, 0.2] | 0.1*** [0.1, 0.1] | 0.1*** [0.1, 0.1] | 0.1*** [0.1, 0.1] |
| *** p<.001, ** p<.01, * p<.025 | | | | | |

**Supplementary Table 7. Cross-sectional, prospective longitudinal, and longitudinal change-based models of the relationship between messaging/ video calling friends and high wellbeing**

|  | Cross-sectional (T2) Weekday | Cross-sectional (T2) Weekend | Prospective longitudinal (T1🡪 T2) Weekday | Prospective longitudinal (T1🡪 T2) Weekend | Longitudinal change (between T1 & T2) |
| --- | --- | --- | --- | --- | --- |
| Main effect chi-square test | **X^2^(3)=10.16, p=0.017** | **X^2^(2)=8.06, p=0.018** | **X^2^(3)=6.05, p=0.109** | **X^2^(2)=0.78, p=0.677** | **X^2^(4)=4.06, p=0.398** |
| Gender |  |  |  |  |  |
| Male | 1.0 | 1.0 | 1.0 | 1.0 | 1.0 |
| Female | 0.4*** [0.3, 0.4] | 0.4*** [0.3, 0.4] | 0.4*** [0.4, 0.5] | 0.4*** [0.4, 0.5] | 0.5*** [0.4, 0.5] |
| SES |  |  |  |  |  |
| Lowest 20% | 1.0 | 1.0 | 1.0 | 1.0 | 1.0 |
| Middle 60% | 1.5*** [1.2, 1.8] | 1.5*** [1.2, 1.8] | 1.4** [1.1, 1.7] | 1.4** [1.1, 1.7] | 1.4** [1.1, 1.7] |
| Upper 20% | 1.8*** [1.4, 2.3] | 1.8*** [1.4, 2.3] | 1.8*** [1.4, 2.4] | 1.8*** [1.4, 2.4] | 1.8*** [1.4, 2.4] |
| Trial group |  |  |  |  |  |
| Control | 1.0 | 1.0 | 1.0 | 1.0 | 1.0 |
| Intervention | 1.2 [1.0, 1.5] | 1.2* [1.0, 1.5] | 1.2* [1.0, 1.5] | 1.2* [1.0, 1.5] | 1.2* [1.0, 1.5] |
| State |  |  |  |  |  |
| NSW | 1.0 | 1.0 | 1.0 | 1.0 | 1.0 |
| QLD | 1.0 [0.8, 1.2] | 1.0 [0.8, 1.2] | 1.0 [0.8, 1.2] | 1.0 [0.8, 1.2] | 1.0 [0.8, 1.2] |
| WA | 0.9 [0.7, 1.2] | 0.9 [0.7, 1.2] | 0.9 [0.7, 1.2] | 0.9 [0.7, 1.2] | 0.9 [0.7, 1.2] |
| Average daily T2 screen time (hrs) | 0.9*** [0.9, 1.0] | 0.9*** [0.9, 1.0] | 0.9*** [0.9, 1.0] | 0.9*** [0.9, 1.0] | 0.9*** [0.9, 1.0] |
| Weekday messaging freq at T2 |  |  |  |  |  |
| Every day | 1.4** [1.1, 1.8] |  |  |  |  |
| 3-4 days | 1.2 [0.9, 1.7] |  |  |  |  |
| 1-2 days | 1.5* [1.1, 2.1] |  |  |  |  |
| Not usually / Never | 1.0 |  |  |  |  |
| Weekend messaging freq at T2 |  |  |  |  |  |
| Both days |  | 1.4** [1.1, 1.9] |  |  |  |
| One day |  | 1.4* [1.0, 1.8] |  |  |  |
| Not usually / Not at all |  | 1.0 |  |  |  |
| T1 High wellbeing |  |  |  |  |  |
| No |  |  | 1.0 | 1.0 | 1.0 |
| Yes |  |  | 5.2*** [4.3, 6.4] | 5.2*** [4.2, 6.3] | 5.2*** [4.3, 6.4] |
| Weekday messaging freq at T1 |  |  |  |  |  |
| Every day |  |  | 1.1 [0.8, 1.4] |  |  |
| 3-4 days |  |  | 0.8 [0.6, 1.1] |  |  |
| 1-2 days |  |  | 1.2 [0.9, 1.6] |  |  |
| Not usually / Never |  |  | 1.0 |  |  |
| Weekend messaging freq at T1 |  |  |  |  |  |
| Both days |  |  |  | 1.1 [0.9, 1.4] |  |
| One day |  |  |  | 1.1 [0.8, 1.4] |  |
| Not usually / Not at all |  |  |  | 1.0 |  |
| Change in messaging between T1 and T2 |  |  |  |  |  |
| large decrease |  |  |  |  | 0.9 [0.7, 1.2] |
| small decrease |  |  |  |  | 1.1 [0.8, 1.4] |
| no change |  |  |  |  | 1.0 |
| small increase |  |  |  |  | 1.2 [0.9, 1.5] |
| large increase |  |  |  |  | 1.2 [0.9, 1.5] |
| Intercept | 0.5*** [0.4, 0.8] | 0.5*** [0.4, 0.7] | 0.3*** [0.2, 0.5] | 0.3*** [0.2, 0.4] | 0.3*** [0.2, 0.5] |
| *** p<.001, ** p<.01, * p<.025 | | | | | |

**Supplementary Table 8. Cross-sectional, prospective longitudinal, and longitudinal change-based models of the relationship between social media posting and high wellbeing**

|  | Cross-sectional (T2) Weekday | Cross-sectional (T2) Weekend | Prospective longitudinal (T1🡪 T2) Weekday | Prospective longitudinal (T1🡪 T2) Weekend | Longitudinal change (between T1 & T2) |
| --- | --- | --- | --- | --- | --- |
| Main effect chi-square test | **X^2^(3)=8.1, p=0.044** | **X^2^(2)=0.43, p=0.808** | **X^2^(3)=0.68, p=0.878** | **X^2^(2)=3.62, p=0.163** | **X^2^(4)=2.02, p=0.733** |
| Gender |  |  |  |  |  |
| Male | 1.0 | 1.0 | 1.0 | 1.0 | 1.0 |
| Female | 0.4*** [0.3, 0.4] | 0.4*** [0.3, 0.4] | 0.4*** [0.4, 0.5] | 0.4*** [0.4, 0.5] | 0.4*** [0.4, 0.5] |
| SES |  |  |  |  |  |
| Lowest 20% | 1.0 | 1.0 | 1.0 | 1.0 | 1.0 |
| Middle 60% | 1.5*** [1.2, 1.8] | 1.5*** [1.2, 1.8] | 1.4** [1.1, 1.8] | 1.4** [1.1, 1.8] | 1.4** [1.1, 1.7] |
| Upper 20% | 1.8*** [1.4, 2.3] | 1.8*** [1.4, 2.3] | 1.8*** [1.4, 2.4] | 1.8*** [1.4, 2.4] | 1.8*** [1.4, 2.4] |
| Trial group |  |  |  |  |  |
| Control | 1.0 | 1.0 | 1.0 | 1.0 | 1.0 |
| Intervention | 1.2* [1.0, 1.5] | 1.2* [1.0, 1.5] | 1.3** [1.1, 1.5] | 1.3** [1.1, 1.5] | 1.3** [1.1, 1.5] |
| State |  |  |  |  |  |
| NSW | 1.0 | 1.0 | 1.0 | 1.0 | 1.0 |
| QLD | 0.9 [0.8, 1.2] | 0.9 [0.8, 1.2] | 1.0 [0.8, 1.2] | 1.0 [0.8, 1.2] | 1.0 [0.8, 1.2] |
| WA | 0.9 [0.7, 1.2] | 0.9 [0.7, 1.2] | 0.9 [0.7, 1.3] | 0.9 [0.7, 1.3] | 0.9 [0.7, 1.3] |
| Average daily T2 screen time (hrs) | 0.9*** [0.9, 1.0] | 0.9*** [0.9, 1.0] | 0.9*** [0.9, 1.0] | 0.9*** [0.9, 1.0] | 0.9*** [0.9, 1.0] |
| Weekday posting freq at T2 |  |  |  |  |  |
| Every day | 1.0 [0.7, 1.6] |  |  |  |  |
| 3-4 days | 0.7* [0.5, 0.9] |  |  |  |  |
| 1-2 days | 1.0 [0.8, 1.3] |  |  |  |  |
| Not usually / Never | 1.0 |  |  |  |  |
| Weekend posting freq at T2 |  |  |  |  |  |
| Both days |  | 1.0 [0.8, 1.2] |  |  |  |
| One day |  | 1.1 [0.8, 1.4] |  |  |  |
| Not usually / Not at all |  | 1.0 |  |  |  |
| T1 High psych distress |  |  |  |  |  |
| No |  |  | 1.0 | 1.0 | 1.0 |
| Yes |  |  | 5.3*** [4.4, 6.5] | 5.4*** [4.4, 6.5] | 5.3*** [4.4, 6.5] |
| Weekday posting freq at T1 |  |  |  |  |  |
| Every day |  |  | 1.1 [0.8, 1.6] |  |  |
| 3-4 days |  |  | 0.9 [0.7, 1.3] |  |  |
| 1-2 days |  |  | 1.0 [0.9, 1.3] |  |  |
| Not usually / Never |  |  | 1.0 |  |  |
| Weekend posting freq at T1 |  |  |  |  |  |
| Both days |  |  |  | 1.3 [1.0, 1.8] |  |
| One day |  |  |  | 1.0 [0.8, 1.3] |  |
| Not usually / Not at all |  |  |  | 1.0 |  |
| Change in posting between T1 and T2 |  |  |  |  |  |
| large decrease |  |  |  |  | 1.0 [0.7, 1.5] |
| small decrease |  |  |  |  | 1.1 [0.9, 1.4] |
| no change |  |  |  |  | 1.0 |
| small increase |  |  |  |  | 0.9 [0.7, 1.3] |
| large increase |  |  |  |  | 1.0 [0.7, 1.4] |
| Intercept | 0.7* [0.5, 0.9] | 0.7* [0.5, 0.9] | 0.3*** [0.2, 0.4] | 0.3*** [0.2, 0.4] | 0.3*** [0.2, 0.4] |
| *** p<.001, ** p<.01, * p<.025 | | | | | |

**Supplementary Table 9. Cross-sectional, prospective longitudinal, and longitudinal change-based models of the relationship between social media viewing and high wellbeing**

|  | Cross-sectional (T2) Weekday | Cross-sectional (T2) Weekend | Prospective longitudinal (T1🡪 T2) Weekday | Prospective longitudinal (T1🡪 T2) Weekend | Longitudinal change (between T1 & T2) |
| --- | --- | --- | --- | --- | --- |
| Main effect chi-square test | **X^2^(3)=6.73, p=0.081** | **X^2^(2)=4.47, p=0.107** | **X^2^(3)=5.78, p=0.123** | **X^2^(2)=0.04, p=0.983** | **X^2^(4)=4.83, p=0.306** |
| Gender |  |  |  |  |  |
| Male | 1.0 | 1.0 | 1.0 | 1.0 | 1.0 |
| Female | 0.4*** [0.3, 0.4] | 0.4*** [0.3, 0.4] | 0.5*** [0.4, 0.5] | 0.4*** [0.4, 0.5] | 0.4*** [0.4, 0.5] |
| SES |  |  |  |  |  |
| Lowest 20% | 1.0 | 1.0 | 1.0 | 1.0 | 1.0 |
| Middle 60% | 1.5*** [1.2, 1.9] | 1.5*** [1.2, 1.9] | 1.4** [1.1, 1.8] | 1.4** [1.1, 1.7] | 1.4** [1.1, 1.8] |
| Upper 20% | 1.8*** [1.5, 2.3] | 1.8*** [1.5, 2.3] | 1.8*** [1.4, 2.4] | 1.8*** [1.4, 2.4] | 1.8*** [1.4, 2.4] |
| Trial group |  |  |  |  |  |
| Control | 1.0 | 1.0 | 1.0 | 1.0 | 1.0 |
| Intervention | 1.2 [1.0, 1.5] | 1.2 [1.0, 1.5] | 1.2* [1.0, 1.5] | 1.2* [1.0, 1.5] | 1.2* [1.0, 1.5] |
| State |  |  |  |  |  |
| NSW | 1.0 | 1.0 | 1.0 | 1.0 | 1.0 |
| QLD | 0.9 [0.8, 1.2] | 0.9 [0.8, 1.2] | 1.0 [0.8, 1.2] | 1.0 [0.8, 1.2] | 1.0 [0.8, 1.2] |
| WA | 0.9 [0.7, 1.2] | 0.9 [0.7, 1.2] | 0.9 [0.7, 1.2] | 0.9 [0.7, 1.2] | 0.9 [0.7, 1.2] |
| Average daily T2 screen time (hrs) | 0.9*** [0.9, 1.0] | 0.9*** [0.9, 1.0] | 0.9*** [0.9, 1.0] | 0.9*** [0.9, 1.0] | 0.9*** [0.9, 1.0] |
| Weekday viewing freq at T2 |  |  |  |  |  |
| Every day | 0.8 [0.6, 1.1] |  |  |  |  |
| 3-4 days | 0.7 [0.5, 1.0] |  |  |  |  |
| 1-2 days | 1.0 [0.7, 1.5] |  |  |  |  |
| Not usually / Never | 1.0 |  |  |  |  |
| Weekend viewing freq at T2 |  |  |  |  |  |
| Both days |  | 0.8 [0.6, 1.0] |  |  |  |
| One day |  | 0.9 [0.7, 1.2] |  |  |  |
| Not usually / Not at all |  | 1.0 |  |  |  |
| T1 High wellbeing |  |  |  |  |  |
| No |  |  | 1.0 | 1.0 | 1.0 |
| Yes |  |  | 5.2*** [4.3, 6.4] | 5.2*** [4.2, 6.4] | 5.2*** [4.2, 6.4] |
| Weekday viewing freq at T1 |  |  |  |  |  |
| Every day |  |  | 1.0 [0.8, 1.2] |  |  |
| 3-4 days |  |  | 1.2 [0.9, 1.7] |  |  |
| 1-2 days |  |  | 0.9 [0.6, 1.3] |  |  |
| Not usually / Never |  |  | 1.0 |  |  |
| Weekend viewing freq at T1 |  |  |  |  |  |
| Both days |  |  |  | 1.0 [0.8, 1.3] |  |
| One day |  |  |  | 1.0 [0.7, 1.4] |  |
| Not usually / Not at all |  |  |  | 1.0 |  |
| Change in viewing between T1 and T2 |  |  |  |  |  |
| large decrease |  |  |  |  | 1.2 [0.8, 1.7] |
| small decrease |  |  |  |  | 1.1 [0.8, 1.5] |
| no change |  |  |  |  | 1.0 |
| small increase |  |  |  |  | 1.2 [0.9, 1.5] |
| large increase |  |  |  |  | 0.9 [0.7, 1.1] |
| Intercept | 0.8 [0.6, 1.2] | 0.8 [0.6, 1.2] | 0.3*** [0.2, 0.5] | 0.3*** [0.2, 0.5] | 0.3*** [0.2, 0.4] |
| *** p<.001, ** p<.01, * p<.025 | | | | | |

1. Coyne, S.M., et al., *Suicide Risk in Emerging Adulthood: Associations with Screen Time over 10 years.* J Youth Adolesc, 2021. **50**(12): p. 2324-2338.

2. Boers, E., et al., *Association of Screen Time and Depression in Adolescence.* JAMA Pediatr, 2019. **173**(9): p. 853-859.

3. Boers, E., M.H. Afzali, and P. Conrod, *Temporal Associations of Screen Time and Anxiety Symptoms Among Adolescents.* Can J Psychiatry, 2020. **65**(3): p. 206-208.

4. Kaur, N., et al., *Associations between digital technology and substance use among U.S. adolescents: Results from the 2018 Monitoring the Future survey.* Drug Alcohol Depend, 2020. **213**: p. 108124.

5. Beyens, I., et al., *The effect of social media on well-being differs from adolescent to adolescent.* Scientific Reports, 2020. **10**(1): p. 10763.
